# Impacts of plastic film mulching on crop yields, soil water, nitrate, and organic carbon in Northwestern China: A meta-analysis

**DOI:** 10.1016/j.agwat.2018.02.001

**Published:** 2018-04-01

**Authors:** Dedi Ma, Lei Chen, Hongchao Qu, Yilin Wang, Tom Misselbrook, Rui Jiang

**Affiliations:** a Key Laboratory of Plant Nutrition and the Agri-environment in Northwest China, Ministry of Agriculture, College of Natural Resources and Environment, Northwest A&F University, Yangling, 712100, China; b Key Laboratory of Bio-Resource and Eco-Environment of Ministry of Education, College of Life Sciences, Sichuan University, Chengdu 610065, China; c Department of Sustainable Soils and Grassland Systems, Rothamsted Research, North Wyke, Devon, Okehampton, EX20 2SB, UK

**Keywords:** Plastic film mulching, Economic benefit, Grain yield, Soil water content, Nitrate

## Abstract

In order to increase crop yield in semi-arid and arid areas, plastic film mulching (PFM) is widely used in Northwestern China. To date, many studies have addressed the effects of PFM on soil physical and biochemical properties in rain-fed agriculture in Northwestern China, but the findings of different studies are often contradictory. Therefore, a comprehensive review of the impacts of PFM on soil water content, soil nutrients and food production is needed. We compiled the results of 1278 observations to evaluate the overall effects of PFM on soil water content, the distribution of nitrate and soil organic carbon, and crop yield in rain-fed agriculture in Northwestern China. Our results showed that PFM increased soil moisture and nitrate concentration in topsoils (0–20 cm) by 12.9% and 28.2%, respectively, but slightly decreased (1.8%) soil organic carbon (SOC) content in the 0–10 cm soil layer. PFM significantly increased grain yields by 43.1%, with greatest effect in spring maize (79.4%). When related to cumulative precipitation during the crop growing season, yield increase from PFM was greatest (72.8%) at 200–300 mm, which was attributed to the large increase for spring maize and potato, implying that crop zoning would be beneficial for PFM in this region. When related to N application rate, crop yields benefited most from PFM (80.2%) at 200–300 kg/ha. A cost-benefit analysis indicated that PFM increased economic return by an average of 29.5%, with the best improvement for spring maize (71.1%) and no increase for spring wheat. In conclusion, PFM can significantly increase crop yield and economic return (especially for spring maize) in rain-fed agriculture areas of Northwestern China. Crop zoning is recommended for PFM to achieve the largest economic benefit. However, full account needs to be taken of the environmental impacts relating to N loss, SOC depletion and film pollution to evaluate the sustainability of PFM systems and further research is required to quantify and mitigate these impacts.

## Highlights

• Our study can provide a comprehensive assessment on the impacts of the plastic film mulching.
• We assessed the effects of plastic film mulching on soil physical and biochemical properties.
• The spring maize yield was significantly increased with plastic film mulching in Northwest China.

## 1 Introduction

As the human population increases, the global demand for food is expected to double by 2050 (Tilman et al., 2012). With decreasing availability of well-watered agricultural lands, existing cropland with limited water supply such as those in rain-fed arid and semiarid areas will need to be used more effectively to attain the required food production levels, (Fischer and Turner, 1978; Haddad et al., 2010). In China, approximately one third of the dryland farming is in the arable land areas, of which about 40% are situated on the Chinese Loess Plateau (Li et al, 2004). Thus the Chinese Loess Plateau has the potential to be a major food production area of China in the 21st century if appropriate agricultural technologies can be applied to solve the water stress issue.

Since plastic film mulching (hereafter refer to “PFM”) can increase the water content of shallow soils, protect soil water from evaporation and improve soil temperature (Ravi and Lourduraj, 1996; Huang et al., 1999), it has been widely applied in areas of the Chinese Loess Plateau to increase crop yields and ensure a sufficient food supply for the growing population (Deng et al., 2006). Many studies have assessed the influence of PFM on the yield of various crops (e.g., maize and wheat) through impacts on soil water content, soil temperature, soil nutrients, and even soil microbes (Cook et al., 2006; Subrahmaniyan et al., 2006). However, the findings of these studies are often contradictory or inconsistent in relation to PFM application in semiarid areas. For example, while PFM is often shown to increase crop yield, reductions in yield have also been observed (Du et al., 2004). Li et al. (1999) reported that PFM reduced spring wheat yield due to low antecedent soil moisture and nutrient depletion during the mulching period. Even where increases in crop yield with PFM have been reported, the reason for the increase or underlying mechanism may differ for different crops or different climatic regions. Some studies have suggested that the mechanism for yield increase under PFM is an improvement in soil water and temperature conditions and an enhancement of soil nutrient availability; although also associated with the consumption of soil organic carbon (Wilson and Jefferies, 1996; Gao et al., 2009). Several studies have observed a decrease in soil organic carbon under PFM due to enhanced soil mineralization (Li et al., 2009; Li and Li, 2015), raising questions regarding sustainability. On the other hand, Liu et al. (2014) and Gao et al. (2014) reported that PFM increased crop root growth and root exudates, thus promoting soil organic carbon accumulation. These differences could be related to different crops with different root systems, differences in the number of years that mulching has been practiced (short vs long-term) or different management practices (i.e. high N input could stimulate soil organic matter mineralization). Therefore, PFM may have negative effects if applied inappropriately, not only decreasing crop yield, but also promoting soil degradation. Wang et al. (2006) showed that PFM resulted in nitrate accumulation in the top soil, potentially decreasing N leaching during storms but increasing greenhouse gas (nitrous oxide) emission. Liu et al. (2014) found that the nitrate accumulation under PFM had a positive relationship with N input. Hence, the effects of PFM on crop yields and agricultural ecosystems are variable, considering the different factors, such as climate (precipitation and temperature), crops, soils, and agricultural management practices (e.g. N input levels), and a comprehensive assessment based on all available data is needed to evaluate the economic and environmental sustainability of the practice for arid and semiarid regions.

The objectives of this study, therefore, were to comprehensively evaluate through meta-analysis the effects of PFM on crop yield, soil water content, and soil nutrients (i.e., soil nitrate and soil organic carbon) under a range of conditions, and the economic benefit of PFM in the rain-fed agriculture areas of Northwestern China.

## 2 Materials and methods

### 2.1 Data

The Web of Science and China National Knowledge Internet were used to find peer-reviewed studies published before January 2017. Search terms included ‘plastic film mulch’ or ‘mulching’, ‘nitrogen’, ‘nitrate’, ‘water content’ or ‘soil organic carbon’ in the article title, abstract, and keywords. The following five criteria were defined for a study to be included in the analysis: i) the field experiment and the experimental sites were located in rain-fed agriculture areas of Northwestern China (Shaanxi; Gansu; Qinghai; Xinjiang; northwest Inner Mongolia); ii) the crop grain was harvested at the physiological mature stage; iii) in addition to the treatment; a control group without the application of PFM was included in the experiment design; iv) reported averages of observational data were based on at least three replicates; v) the application rates of nutrient inputs (fertilizer N; P and K) were reported; for inclusion in the cost–benefit analysis. Accordingly; a total of 1278 observations from 83 peer-reviewed studies were included in our analysis.

### 2.2 Effect size

To quantify the impacts of PFM on a given variable, the response ratio (*R*) was determined according to Hedges et al. (1999):

(1) $$\ln R=\ln\left(\frac{Xt}{Xc}\right),$$

where *X_t_* and *X_c_* are the treatment value (i.e. under PFM) and corresponding control value, respectively, for the given variable. The results were presented as the percentage change ((R-1) × 100) under PFM, with a positive percentage change denoting an increase in variable value due to PFM and a negative value denoting a decrease.

Effect sizes can be weighted using the inverse of the pooled variance (Yang et al., 2016) or the number of replications (Lam et al., 2012), depending on the integrity of the reported standard deviations in the database. Over 50% of the studies included in our meta-analysis did not report the standard deviations of the mean values. In addition, extreme weights may be generated using variance-based weighting functions, but not when using replication-based approaches (Van Groenigen et al., 2011). Therefore, the replication based weighting was adopted in our analysis using the following equation (Lam et al., 2012):

(2) $$\text{weight}=\frac{nt\times nc}{nt+nc},$$

where *n_t_* and *n_c_* represent the numbers of replicates of the treatment and control groups, respectively. Mean effect sizes and the 95% confidence intervals (CIs) were generated by a bootstrapping procedure with 4999 iterations, using METAWIN 2.1 (Rosenberg et al., 2000). Effects of PFM were considered significant if the 95% CIs did not overlap with zero. Similarly, means of categorical variables were considered significantly different from each other if their 95% CIs did not overlap (Xia et al., 2017).

### 2.3 Cost-benefit analysis

Cost-benefit analysis included assessment of the input costs, income from yield sales and net economic benefit (NEB). The input costs included the cost of agricultural materials such as seed, fertilizer, pesticides, and plastic film (http://www.npcs.gov.cn/and http://china.guidechem.com/), and labor cost associated with fertilizer/pesticide applications and mechanical operations (Table S1). Yield income refers to income from grain yield. The NEB was calculated by subtracting the input cost from the yield income (Xia et al., 2017).

## 3 Results

### 3.1 Effect of PFM on soil water content

**Fig. 1:**
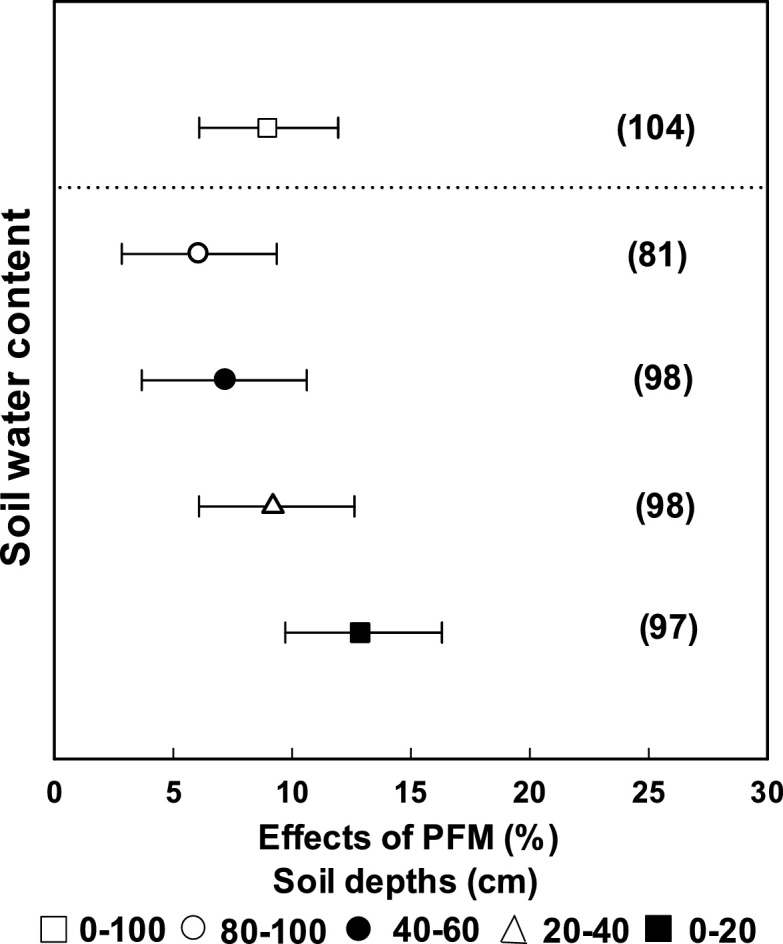
Changes in soil water content affected by PFM at different soil depths. The numbers in parentheses indicate the number of observations.

On average, PFM increased soil water content by 9.0% across all soil layers (Fig. 1), compared with traditional cultivation. The effect decreased with increasing soil depth; for example, soil water content at 0–20 cm depth was increased by 12.9%, more than twice of that at 80–100 cm depth (6.1%) (Fig. 1).

### 3.2 Effect of PFM on soil nitrate and soil organic carbon distribution

**Fig. 2:**
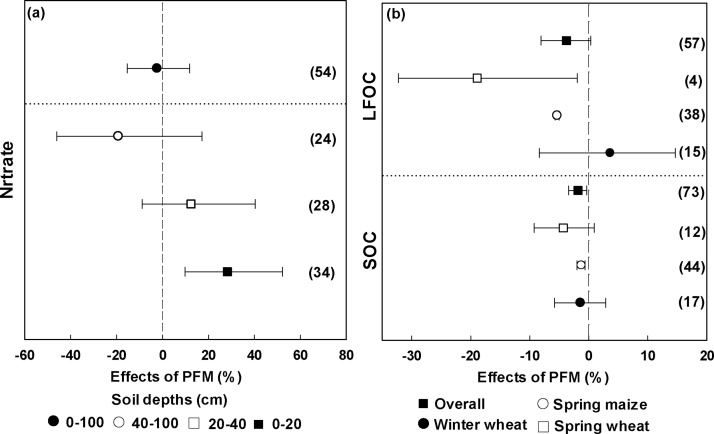
Changes in soil (a) nitrate and (b) carbon at different depths with PFM. The numbers in parentheses indicate the number of observations.

Overall, PFM had no significant effect on soil nitrate at 0–100 cm soil depth (Fig. 2a). However, with PFM, soil nitrate content was significantly increased by 28.2% in the 0–20 cm soil layer (Fig. 2a).

The soil organic carbon (SOC) contents of the 0–10 cm soil layer were slightly decreased (1.8%), although changes in the light fraction organic carbon (LFOC) in surface soil (0–10 cm)were not significant under PFM across all crops in Northwestern China (Fig. 2b). However, the LFOC contents in topsoils were significantly decreased for spring wheat and spring maize (Fig. 2b).

### 3.3 Effect of PFM on grain yield

#### 3.3.1 Effect of PFM on grain yield and water use efficiency (WUE) for different crops

**Fig. 3:**
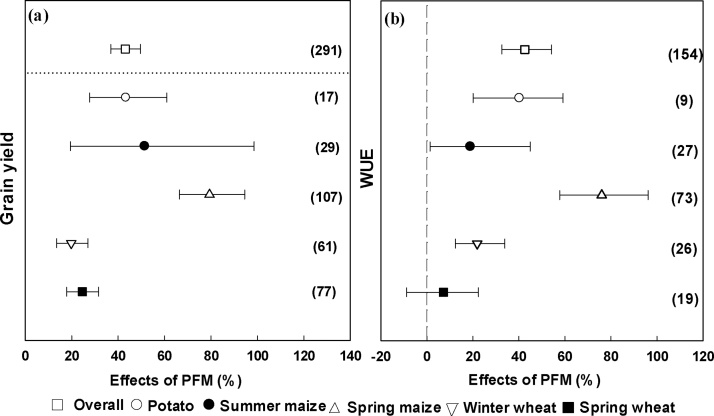
Changes in (a) grain yield and (b) WUE induced by PFM for different crops. The numbers in parentheses indicate the number of observations.

Overall, compared with traditional cultivation, grain yield was significantly increased by 43.1% with PFM (Fig. 3a). The yield increase in maize, including spring maize (79.4%) and summer maize (51.4%) was greater than that of potato (43.4%) and wheat (19.8% and 24.6% for winter wheat and spring wheat, respectively) (Fig. 3a).

The average WUE across all crops was significantly increased by 42.6% with PFM (Fig. 3b), with the highest increase in spring maize (76%), followed by potato (40.2%), winter wheat (21.9%) and summer maize (18.9%) (Fig. 3b). However, the PFM had no significant effect on the WUE of spring wheat (Fig. 3b).

#### 3.3.2 Effect of PFM on grain yield for different cumulative precipitation (in the growing season)

**Fig. 4:**
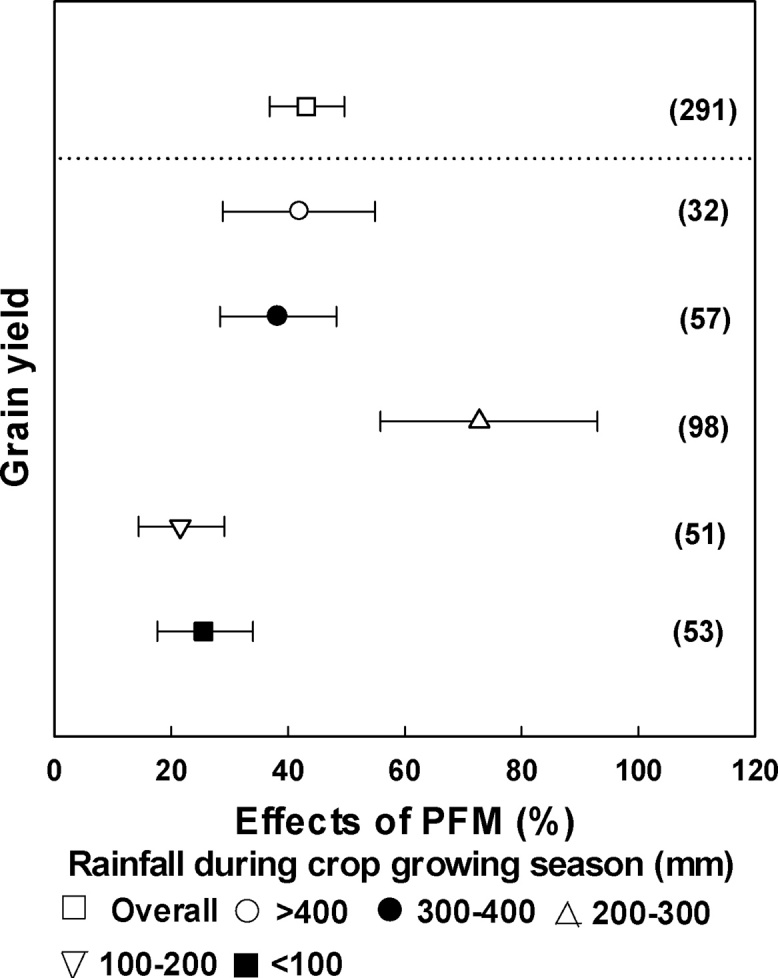
Changes in grain yield affected by PFM under different cumulative rainfall during the growing season. The numbers in parentheses indicate the number of observations.

The effect of the PFM on crop yield was significantly different under different rainfall conditions during growing season (Fig. 4). The increase in crop yield was greatest (72.8%) when cumulative rainfall in the growing season was in the range 200–300 mm (Fig. 4). The yields were increased by 38.3–42.0% and 21.5–25.5%, respectively, when the cumulative rainfall was >300 mm and < 200 mm (Fig. 4).

**Fig. 5:**
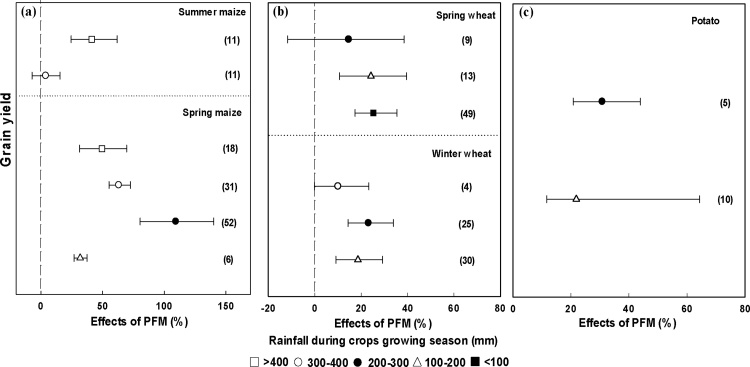
Changes in grain yield affected by PFM under different cumulative rainfall over the growing season for maize (a), wheat (b) and potato (c). The numbers in parentheses indicate the number of observations.

The yield of summer maize was significantly increased by 42.0% when the cumulative rainfall during the growing period was > 400 mm, whereas there was no significant effect of PFM when cumulative rainfall was 300–400 mm (Fig. 5a). For spring maize, PFM had a greater effect on grain yield for cumulative rainfall in the range 200–300 mm (109.4%) than for other rainfall amounts (Fig. 5a). PFM increased spring wheat yield by 22% across all cumulative rainfall amounts, with the highest increase (25.2%) when cumulative rainfall was less <100 mm (Fig. 5b). For winter wheat, greatest effect (23.1%) was for cumulative rainfall of 200–300 mm (Fig. 5b). For potato, a greater effect on yield under PFM was observed when cumulative rainfall was 200–300 mm (30.8%) compared to 100–200 mm (21.9%) (Fig. 5c).

#### 3.3.3 Effect of PFM on grain yield under different N application rates

**Fig. 6:**
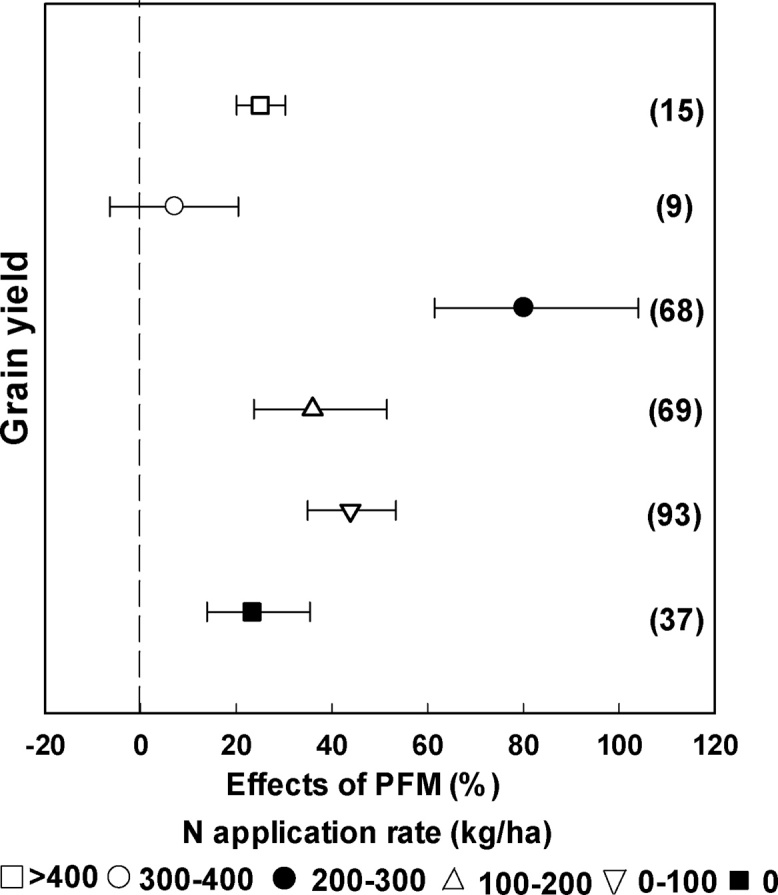
Changes in grain yield affected by PFM for different N application rates. The numbers in parentheses indicate the number of observations.

Across all crops, PFM enhanced crop yield at all N fertilizer application rates (Fig. 6), with greatest effect at 200–300 kg/ha (80.2%). For N application rates >300 kg/ha, the effect of PFM on enhancing grain yield decreased dramatically (Fig. 6). It is worth noting that PFM significantly increased crop yield at zero N input (23.3%) (Fig. 6).

**Fig. 7:**
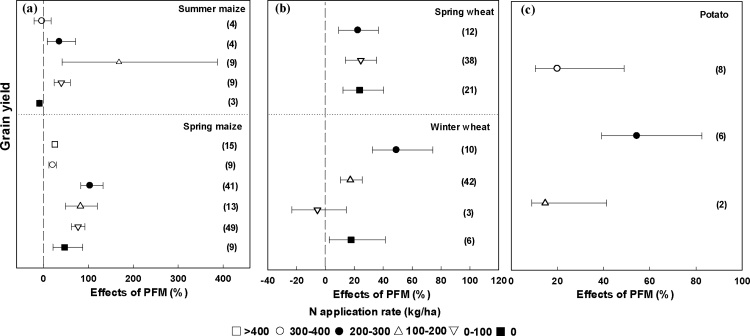
Changes in grain yield affected by PFM at different N application rates for different crops. The numbers in parentheses are indicate the number of observations.

For summer maize, the greatest impact of PFM on yield was 168.1% at a low N input (100–200 kg/ha) (Fig. 7a), with no significant effect at N applications of 300–400 kg/ha (Fig. 7a). Noticeably, summer maize yield was significantly reduced under PFM by 9.3% if there was no N input (Fig. 7a). For spring maize, the greatest yield increase with PFM was 103.3% at N application rates of 200–300 kg/ha. The effect of PFM on grain yield was 82.2%, 76.9% and 46.8% at low N rates (100–200 kg/ha, 0–100 kg/ha, 0 kg/ha) and 25%, 20.2% at high N input (>400 kg/ha, 300–400 kg/ha) (Fig. 7a). For spring wheat, yield increase with PFM was similar (23.5%)across the different N application rates (Fig. 7b). Interestingly, the effect of PFM on winter wheat yield became non-significant at N rates of 0–100 kg/ha (Fig. 7b). Moreover, the greatest yield increase with PFM was 49.1% at N application rates of 200–300 kg/ha (Fig. 7b). The greatest yield effect of PFM for potato was 54.4% at N application rates of 200–300 kg/ha (Fig. 7c).

### 3.4 Effect of PFM on net economic benefit

**Fig. 8:**
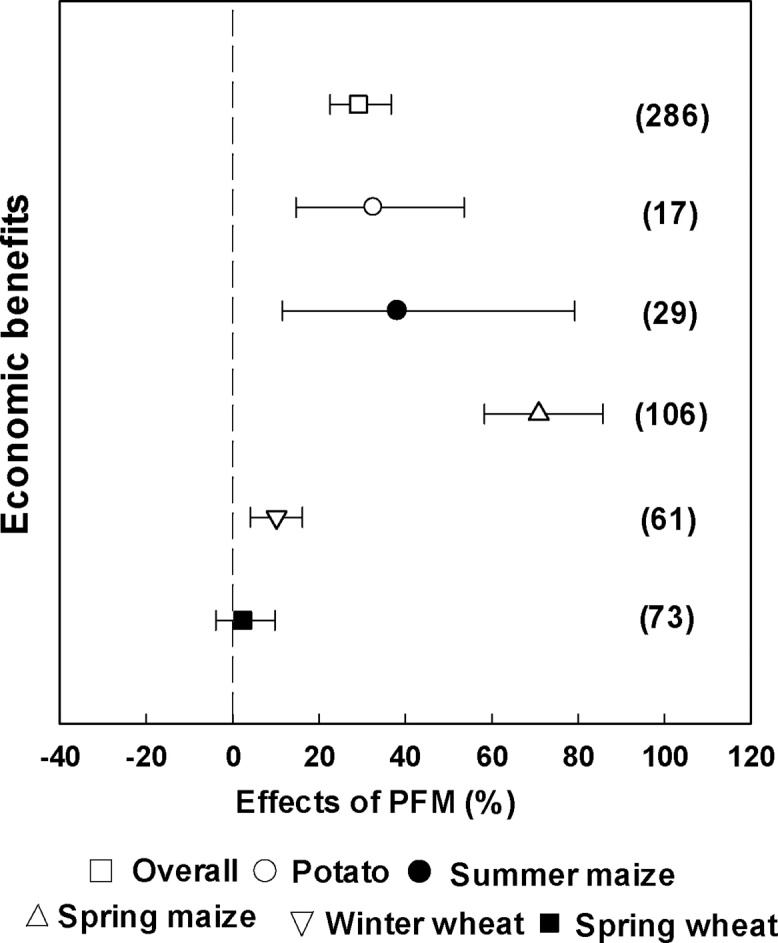
Economic benefit analysis for different crops under PFM. The numbers in parentheses indicate the number of observations.

Compared with traditional cultivation, the average NEB was significantly increased by 29.5% through PFM (Fig. 8). The increased NEB with PFM was greatest in spring maize (71.1%), followed by summer maize (38.6%), potato (33.0%), and winter wheat (10.4%) (Fig. 8). For spring wheat, there was no significant difference in NEB between PFM and traditional cultivation (Fig. 8).

## 4 Discussion

### 4.1 Soil water content

Water is one of the most important driving factors of agricultural production (Hanjra and Qureshi, 2010). We found that PFM increased the soil water content significantly (12.9%) for topsoils (0–20 cm), compared with traditional cultivation, as has also been reported by others (Li et al., 2011; Wang et al., 2009; Liu et al., 2014). The PFM directly inhibits evaporation of water from the soil surface, promotes water movement from deeper soil layers to the topsoil by vapor transfer, and enhances the topsoil water content during critical stages of crop growth (Gan and Siddique, 2013). Our results show that this enhancement in soil water content is not only for topsoils, but for the 0–1 m soil layer (Fig. 1), implying an improvement in soil water storage in the soil profile by PFM. However, there were too few data for soil layers below 1 m to include in the meta-analysis. A previous study showed that PFM might cause a soil water deficit in deep soil layers (Zhang et al., 2011). Meanwhile, Zhang et al. (2007) showed that water reached deeper horizons under mulching, resulting in 15% more deep percolation in a wet year. Thus, any water deficit might be recharged during wet year. The effect of PFM on deeper soil water content is still unclear and should be a focus of future studies.

### 4.2 Soil nitrate and carbon content

The contents of nitrate, SOC, and LFOC in soil are important indicators of soil fertility (Liu et al., 2013). Our study showed that PFM was effective in increasing the nitrate content (28.2%) of topsoils (0–20 cm) and slightly decreased nitrate content in deeper soil layers (but not significantly) (Fig. 2). Gao and Li (2009) suggested that enhanced soil mineralization might be the main reason for an increased nitrate content in topsoils, as a result of higher soil temperature and water content under PFM (Wilson and Jefferies, 1996). In our study, it should be noted that SOC decreased slightly in the 0–10 cm soil layer and that the decrease in LFOC content in topsoil was significant for spring maize and spring wheat under PFM (Fig. 2). The amount of organic C in the soil is dependent upon the rate of organic matter decomposition and the amount of crop residue returned to the soil (Liang et al., 2010). PFM likely enhances soil organic matter decomposition, through increased water content and heat in the surface soil (Song et al., 2002), enhancing soil microbial activity, thus promoting microbial degradation of soil organic carbon (Pang and Huang, 2006). However, Liu et al. (2014) note that the dynamics of SOC (FLOC) can relate to the number of years that mulching has been practiced and that long-term mulching may increase SOC (FLOC) through an increase in root biomass.

### 4.3 Grain yield

The Northwest region is an important food production area in China. Our study showed that PFM significantly increased grain production by an average of 43.1% (19.8%–79.4%) in Northwestern China (Fig. 3a). Our analysis confirmed findings of previous studies that PFM is an effective cultivation practice to improve food production in rain-fed agricultural areas (Jiang et al., 2016; Zhang et al., 2014). This increase in yield was largely attributed to improved WUE (Fig. 3b) and a more suitable soil environment for crop growth, because PFM can improve the soil water content (Wang, 1998), increase soil temperature, enhance the activation of soil nutrients, improve soil nutrient availability (Zhao et al., 2002; Zhou, 1996), and shorten the growth period of crops. Holding water in the soil and increasing WUE are key drivers for improved crop yield in areas where evaporation is larger than precipitation. The water stress in arid and semiarid rain-fed agriculture areas of Northwestern China is not caused by a lack of total rainfall but by the irregular distribution of rainfall over time and the inefficient management of rainwater (Lin et al., 2015). Thus, the positive effect of PFM on soil water content during water stress periods plays an important role in increasing crop yield, which has been accepted as the primary mechanism of the yield enhancing effect of PFM. For example, many studies noted the largest increase in soil water content with PFM was during the early stage of the maize growing season, which contributed to the improvement of maize production in Loess Plateau (Liu et al., 2009, Liu et al., 2013 ; Zhang et al., 2013; Jiang et al., 2016). However, PFM increased the soil water content by only 9% on average, which could not explain the 43.1% yield increase. Moreno and Moreno (2008) found that PFM enhanced crop nitrogen use efficiency (NUE) as well as soil water content. Wei et al. (2015) showed that PFM significantly increased NUE for wheat and maize, and that NUE was significantly and positively related to soil organic matter. Hence, increased NUE under PFM might be another reason for the increase in crop yield. However, some studies have pointed out that crop yield increases under PFM were based on the consumption of soil organic carbon, which may have negative effects on the soil ecosystem and not be sustainable (Wilson and Jefferies, 1996; Gao and Li, 2009). Our analysis also showed a slight decrease in SOC under PFM and an accumulation of nitrate in topsoils (Fig. 2), which may be of importance regarding the ideal ratio of soil N and C. Therefore, the N and C transformation processes play important roles in both crop yield response and agricultural sustainability, and thus further studies to better quantify these processes are needed in the rain-fed agriculture area of Northwestern China.

The effects of PFM on yield differed for different crops, with increases ranging from 19.8% to 79.4% (Fig. 3a). Averaged across all studies, we found that the yield increasing effect on spring maize (79.4%) was significantly higher than for other crops. On the one hand, this may be attributed to the fact that maize, as a C4 crop, is more photosynthetically efficient (Long et al., 2006) and more sensitive to water deficit; on the other hand, spring maize often suffers from drought and low temperature during the early stage of the growing season (Jiang et al., 2016; Zhang et al., 2014) and thus PFM could play an important role in early stage growth and subsequent increase in grain yield.

Our analysis showed that cumulative rainfall over the crop growing season had significant effect on the crop yield increase observed under PFM and that the effects were different for different crops (Fig. 4, Fig. 5). A notable yield increase was observed (72.8%, Fig. 4) for cumulative growing season rainfall of 200–300 mm, which was attributed to the effects for spring maize and potato (Fig. 5). The reason for the large yield effect for spring maize and potato was that the rainy season for the region coincided with the major crop growing period for these crops, and PFM improved the soil water content and temperature during the early stages (drought period) (Zhang et al., 2014; Jiang et al., 2016). The effect of PFM on summer maize yield was significantly enhanced when the rainfall during the growing period was >400 mm (Fig. 5a). Summer maize grows during the summer period when temperature and evaporation are high, but PFM may further increase temperature, thus inhibiting crop growth under low rainfall (Li et al., 2003). For spring wheat, it seems that the effect of PFM on yield tended to decrease with increased rainfall. The yield increased the largest by 25.5% when the rainfall was less than 100 mm (Fig. 5b), in part this is due to that the rainfall meets the demand of spring wheat is relatively low and it's sensitive to drought when rainfall is lower than 100 mm. For winter wheat, it’s different with spring wheat, which the rainfall of 200–300 mm showed the highest increase for grain yield (Fig. 5b). It's likely because that the winter wheat has longer growth period and the precipitation is mainly in the form of snow, which caused the low utilization rate of precipitation in winter. Due to the uneven distribution of precipitation in Northwestern China and the different impact for different crops, crop zoning should be considered to match the rainfall and crop water requirement under PFM. A recommended crop zoning under PFM from west to east on the Loess Plateau would be spring wheat, potato, spring maize, winter wheat, and summer maize. In particular, regions with cumulative rainfall 200–300 mm during May to September should grow potato or maize to achieve the largest increase of crop yield under PFM.

The effects of PFM on crop grain yield are also affected by N application rate (Fig. 6). Farmers often use excessive N fertilizer to pursue high yields and profits, particularly in China (Ju et al., 2009). However, N losses to the environment can be substantial when the availability of soil N exceeds crop N demand (Cui et al., 2013), and excessive fertilization can lead to declining production, damage the natural environment, and economic waste (Reeves, 1997). Our analysis showed that PFM resulted in the largest grain yield enhancement when the N application rate was 200–300 kg/ha, and there was no significant yield increase at N application rates of 300–400 kg/ha. For most of the crops included in this study, therefore, N application rates of 200–300 kg/ha are recommended for high yields under PFM. This result is consistent with Zhang et al. (2008). We speculate that “N was no longer limiting” was responsible for the large increase in grain yield (Liu et al., 2014). Our result showed that N application rates ranging from 200 to 300 kg/ha nearly satisfied the maize/potato N requirements. Chen et al. (2011) showed that synchronizing the N supply with crop N demand is crucial to improving crop yields. The N application rates are comparable to the rates recommended for high-yielding maize in China (237 kg N/ha on average, Chen et al., 2011). However, for summer maize and spring wheat, the effect of the PFM on yield did not increase with increased N input, implying that factors other than N input were more important in driving the increasing in crop yield under PFM. Interestingly, we found that PFM can significantly increase crop yield by 23.3% at an N application rate of 0 kg/ha (Fig. 6). This may be explained by improved soil moisture but also through enhanced soil fertility. PFM can improve the soil water content and temperature, which may increase the soil microbial activities and enhance the soil mineralization rate (Wang et al., 2006), thereby improving plant N uptake from the soil and subsequent crop yield even with zero fertilizer N input. Thus, PFM may directly influence soil fertility when compared with traditional cultivation. However, it is important to consider potential environmental impacts of the N rates observed to give greatest yield increases under PFM. Few studies have addressed the potential impacts of nitrate accumulation in the soil profile under PFM system (Liu et al., 2014), thus the appropriate recommended N application rate under PFM should be further studied taking account of crop yield and environmental effects.

### 4.4 Economic benefits

**Fig. 9:**
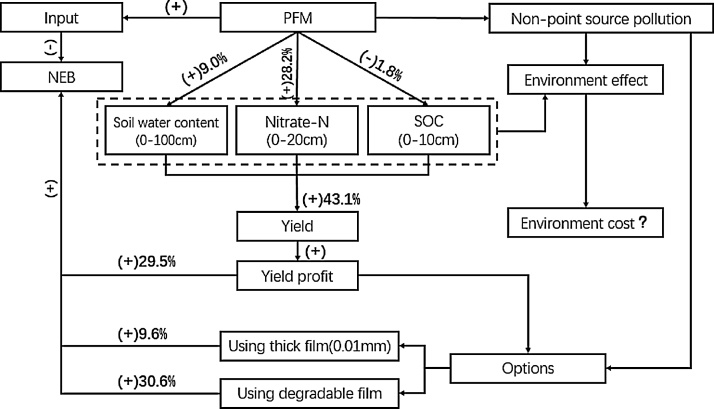
Effects of PFM cultivation on crop productivity, soil water content, soil nitrate and net economic benefit (NEB).

By taking the costs of various agricultural inputs (e.g., fertilizers and mechanical operations) into consideration, we conducted a preliminary assessment of the NEB associated with the implementation of PFM. Although grain yields were significantly enhanced by PFM, the input costs increased accordingly and NEB differed for different crops. Although overall NEB was significantly increased by 29.5% (Fig. 9), for spring wheat was no significant increase. Zhang et al. (2013) showed that wheat production was more likely related to the antecedent soil moisture (before sowing) which was influenced by the previous year's precipitation. Li et al. (1999) found that PFM reduced spring wheat yield because of low antecedent soil moisture and severe nutrient depletion during the mulching period. Therefore, careful consideration should be given to the implementation of PFM for spring wheat in Northwestern China. The impact of PFM on NEB for spring maize was the greatest, suggesting that Northwestern China could be a maize belt if supported by widespread implementation of PFM.

As discussed above, PFM can increase the nitrate content in the topsoil layer, improve NUE and contribute to the yield increase. Additionally, it might reduce N leaching to deeper soil layers (Ruidisch et al., 2013), but may increase N_2_O emissions (Li et al., 2003). There are also potentially negative effects on soil quality, through loss of SOC. However, there were insufficient data available on potential environmental costs to include in the meta-analysis for the NEB and further studies on the environmental influence related to N losses and soil fertility are recommended. In addition, the PFM system showed serious non-point source pollution caused by plastic film (Yan et al., 2006). Use of a biodegradable film or thick (0.01 mm) film may be options to achieve economic benefit and minimise plastic film pollution (Fig. 9).

## 5 Conclusions

Although PFM has been widely applied in arid and semiarid regions such as in Northwestern China to increase the crop production, the impacts of the PFM on crop yields and the underlying mechanisms are still under debate. Here we conducted a comprehensive meta-analysis on the effects of the PFM on three major crops: maize, wheat and potato in Northwest China. To clarify the mechanisms underlying the changes in crop yields, we further assessed how PFM influenced soil biochemical properties, including soil water, nitrate, and SOC (LFOC), and how PFM affected yields under different levels of precipitation and N application rate. Our study suggests that the PFM can significantly increase crop yields, especially spring maize, in Northwestern China. Although the environmental costs of PFM are still unclear due to a lack of data, some options such as using thick film (0.01 mm) or biodegradable film to avoid non-point source pollution may enable high NEB. Our study provides evidence that PFM can be a key practice to increase crop productivity and achieve better economic and environmental benefits in the rain-fed agriculture of Northwestern China. However, the future studies are needed to fully quantify environmental costs, economic befits and agricultural sustainability, particularly relating to N and C transformation processes.
