# Supplementary material for: Impacts of plastic film mulching on crop yields, soil water, nitrate, and organic carbon in Northwestern China: A meta-analysis
Source: Agric Water Manag. 2018 Apr 1;202:166–73. doi: 10.1016/j.agwat.2018.02.001 (PMC5890387; doi:10.1016/j.agwat.2018.02.001)
Supplement: Supplementary file 1 [file mmc1.docx]

Table 1. The price of grains and various agricultural inputs

| Items | Price or cost |
| --- | --- |
| urea | 1.6￥/kg |
| Plastic film (thin, <0.01 mm) | 468￥/hm^2^ |
| Plastic film (thick, 0.01 mm) | 1470￥/hm^2^ |
| Plastic film (biodegradable) | 448.5￥/hm^2^ |
| Mechanical cost | 375￥/hm^2^ |
| Diammonium phosphate | 2.5￥/kg |
| Calcium superphosphate | 1.2￥/kg |
| Potassium sulphate | 4￥/kg |
| Potato | 1.2￥/kg |
| Maize | 2￥/kg |
| Wheat | 2.4￥/kg |
